# New Insights into Cutaneous Asepsis: Synergism between Pfaffia and Rosemary Extracts

**DOI:** 10.3390/antibiotics13030226

**Published:** 2024-02-28

**Authors:** Diego Garcia Miranda, Florence Carrouel, Tatiane Cristina Alberti Silva, Mariana Cafalchio Rozzatto, Amjad Abu Hasna, Carlos Eduardo Rocha Santos, Flavia Villaça Morais, Luciane Dias de Oliveira, Lucas de Paula Ramos

**Affiliations:** 1 Gynecology and Obstetrics Service, Woman-Mother-Child Hospital, Hospices Civils de Lyon, 59 Boulevard Pinel, 69500 Lyon, France; 2 Laboratory Health Systemic Process (P2S), UR4129, Faculty of Medicine Laenenc, University Claude Bernard Lyon 1, University of Lyon, 11 Rue Guillaume Paradin, 69008 Lyon, France; florence.carrouel@univ-lyon1.fr; 3 Multimaterials and Interfaces Laboratory, CNRS (UMR 5615), University Claude Bernard Lyon 1, 6 Rue Victor Grignard, 69622 Lyon, France; 4 Department of Biosciences and Oral Diagnosis, Institute of Science and Technology, São Paulo State University, Francisco José Longo 777, São José dos Campos 12245-000, São Paulo, Brazil; tatiane.alberti@unesp.br (T.C.A.S.); mari_rozzatto@unesp.br (M.C.R.); d.d.s.amjad@gmail.com (A.A.H.); carlos.rocha-santos@unesp.br (C.E.R.S.); luciane.oliveira@unesp.br (L.D.d.O.); 5 Department of Health Sciences, Paulista University, Highway President Dutra, São José dos Campos 12240-420, São Paulo, Brazil; flavia@univap.br; 6 School of Dentistry, Espiritu Santo University, Avenue Samborondon 5, Samborondón 092301, Ecuador; 7 Laboratory of Cell and Molecular Biology of Fungi, Research and Development Institute, Paraíba Valley University, Avenue Shishima Hifumi 2911, São José dos Campos 12244-010, São Paulo, Brazil

**Keywords:** skin infections, herbal medicine, *Staphylococcus aureus*, *Cutibacterium acnes*, *Staphylococcus epidermidis*

## Abstract

(1) Background: In hospitals, medical and dental clinics, antiseptics or disinfectants play an essential role in the control of nosocomial infections. This study aimed to evaluate *R. officinalis* and *P. paniculata* glycolic extracts regarding: (I) their antimicrobial action on planktonic and biofilm (monotypic and cutaneous biofilm model—*S. aureus*, *S. epidermidis* and *C. acnes*); and (II) their cytotoxicity on human keratinocytes (HaCaT). (2) Methods: Minimum inhibitory concentration (MIC) and minimum bactericidal concentration (MBC) were performed (CLSI protocol M7-A6 and M11-A8). MTT analysis was used to evaluate the antibiofilm activity of the extracts on biofilms and their cytotoxicity on human keratinocytes. (3) Results: The combined glycolic extracts MIX A (75% *P. paniculata* + 25% *R. officinalis)*; MIX B (50% *P. paniculata* + 50% *R. officinalis*); and MIX C (25% *P. paniculata* + 75% *R. officinalis*) promoted MBC values by 50 mg/mL on *S. aureus*, absent on *S. epidermidis*, and ranged 6.25–50 mg/mL for *C. acnes.* The cutaneous biofilm model was reduced more than 90%. In addition, it showed biocompatibility with human keratinocytes, resulting in percentages of viability greater than 50%. (4) Conclusions: The combination of extracts promoted antimicrobial action on planktonic cultures, and monotypic and heterotypic biofilms of skin pathogens. Additionally, these extracts are biocompatible against human keratinocytes.

## 1. Introduction

Nosocomial infections, also referred to as healthcare-associated infections, represent a significant burden and safety issue for patients in developing countries. The overall prevalence of healthcare-associated infections in developing countries is up to 15.5%, while it reaches 34.1% for critical care patients [1,2,3,4]. Several Gram-positive bacteria, resident human flora, have been identified as opportunistic bacteria responsible for healthcare-associated infections. Among these bacteria, three appear as very important: (i) *Staphylococcus aureus*, (ii) *Staphylococcus epidermidis*, and (iii) *Cutibacterium acnes* also known as *Proprionibacterium acnes* [5,6]. The *Staphylococcus* genus stands out due to the number of clinical isolates resistant to oxacillin, exhibiting a frightening 81.33% rate of resistance [1].

In hospitals and medical and dental clinics, the appropriate use of gloves is crucial for preventing the transmission of infections from the patient to the medical staff and vice versa. Regardless of the material of the glove used, glove perforations are common in the clinical setting. It has been documented that glove perforation rates during surgery can be as high as 30% [7]. Therefore, antiseptics or disinfectants play an essential role in the control of nosocomial infections, in which antisepsis of skin sites that will receive some type of invasive procedures, including surgical incisions and catheter insertions, are of great importance because of their antimicrobial action, preventing infections of deeper sites [8,9]. Chlorhexidine digluconate (CHX) is a widely used potent disinfectant; however, its continuous use can impose selective pressure on bacteria, promoting the creation of adaptive resistance of these microorganisms. The lack of response of nosocomial pathogens to conventional treatments has become one of the most serious global public health threats of the 21st century, as antimicrobial resistance promotes high rates of mortality and morbidity [10,11,12].

In this scenario, there is a need to search for new, effective, safe, and low-cost alternatives. Phytotherapy is a field of alternative medicine that uses plants to treat diverse diseases, and it is becoming more common in modern medicine because of its antimicrobial action and biocompatibility; for example, *Rosmarinus officinalis*, which is popularly known as rosemary, has phytocompounds that have several pharmacological effects, including antimicrobial, anti-inflammatory, and antioxidant effects [5,13,14,15]. Additionally, *Pfaffia paniculata*, which is a plant native to the Neotropics known as Brazilian ginseng, has antimicrobial, anti-inflammatory, anticancer and regenerative properties [16].

Therefore, the aim of this study was to evaluate *R. officinalis* and *P. paniculata* glycolic extracts regarding (I) their antimicrobial action on planktonic and biofilm (monotypic and heterotypic) cultures of *S. aureus*, *S. epidermidis*, and *C. acnes*; and (II) their cytotoxicity on human keratinocytes (HaCaT) seeking the development of new drugs to combat nosocomial infections.

## 2. Results

### 2.1. Minimum Inhibitory, Minimum Bactericidal, and Fractional Inhibitory Concentrations of P. paniculata and R. officinalis

Table 1 presents the minimum inhibitory concentration (MIC), minimum bactericidal concentration (MBC), and fractional inhibitory concentration (FIC) of *P. paniculate*, *R. officinalis*, and the combined extract on planktonic cultures of *S. aureus*, *S. epidermidis*, and *C. acnes.* The MIC values of all the extracts tested were in the range of 25 to 50 mg/mL in *S. aureus* and *S. epidermidis*, but in the range of 0.195 to 6.25 mg/mL in *C. acnes*. On the other hand, the minimum bactericidal concentration (MBC) values ranged from absent to 50 mg/mL in *S. aureus*. It was absent in *S. epidermidis* and ranged from 6.25 to 50 mg/mL in *C. acnes.* The FIC values of Mix A, B, and C were indifferent in *S. aureus* and were antagonistic in *S. epidermidis*. However, the FIC values of Mix A and B were synergistic, and Mix C was indifferent in *C. acnes*.

### 2.2. Biofilm Viability Measured by MTT Analysis

The percentages of *S. aureus* and *S. epidermidis* biofilm treated during 24 h by several mixtures of *P. paniculata* and *R. officinalis* by MTT analysis is presented in Table 2. On *S. epidermidis*, all the extracts were effective in the reduction of biofilm and had a statistically significant difference compared to the control group (*p* < 0.0001). On *S. aureus*, all the extracts were effective in biofilm reduction, except for *P. paniculata* and Mix A 50 mg/mL. The groups Mix A, Mix B, and Mix C 100 mg/mL were the most effective in biofilm reduction, with a statistically significant difference compared to CHX 0.12%.

The reduction of *C. acnes* biofilm and heterotypic biofilms (in %) by MTT analysis after treatment by the groups for 24 h is shown in Table 3. On *C. acnes*, Mix A 50 and 100 mg/mL were the most effective in biofilm reduction, being statistically as effective as CHX 0.12%. On the cutaneous biofilm model (heterotypic biofilms), all the tested extracts were as effective as CHX 0.12% and had a statistically significant difference compared to the Brain-Heart-Infusion (BHI) control group (*p* < 0.0001). The biofilm reduction varied from 88.73% to 93.50% with the tested extracts.

### 2.3. Scanning Electron Microscopy Analysis

**Figure 1:**
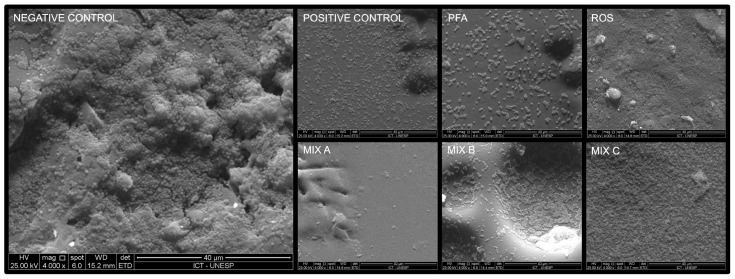
The microbial biofilms in scanning electron microscopy. (Brain heart infusion broth (Negative control), chlorhexidine digluconate 0.06% (Positive control), *P. paniculata* (PFA), *R. officinalis* (ROS), *P. paniculata* and *R. officinalis* 75% + 25% (Mix A), *P. paniculata* and *R. officinalis* 50% + 50% (Mix B), *P. paniculata* and *R. officinalis* 25% + 75% (Mix C). The micrographs were taken at a magnification of 4000×).

It was found that Mix A promoted a great structural reduction of the heterotypic biofilm, leaving only a few cocci deposited on the polystyrene disk. On the other hand, Mix B obtained a greater number of cocci adhering to the surface, visually similar to that obtained by the 0.06% CHX group (positive control). Mix C exhibited high microbial growth (Figure 1).

### 2.4. Cell Viability Measured by MTT Analysis

The application of the extracts on HaCaT resulted in percentages of viability greater than 50%. There was a statistically significant difference between the all the groups and the DMEM + 10% FBS control group (*p* < 0.05). In addition, the *R. officinalis* 100 mg/mL group was as biocompatible as the CHX group (*p* < 0.05). The CHX group promoted a 14.6% cell viability applied for 24 h, resulting in greater cytotoxicity with a statistically significant difference compared to all the tested extracts (*p* < 0.05) (Table 4).

## 3. Discussion

Anaerobic bacteria normally reside on human skin, in the oral cavity, and in the upper respiratory tract, and their removal during surgical procedures is a challenge. In the literature, Foster et al. [17] and Dörfel et al. [18] have proved that bacteria such as *C. acnes* and *S. aureus* have presented high levels of resistance to CHX, so the creation of new antiseptics for prolonged preparations of human skin with agents capable of penetrating deeper is necessary to access mainly *C. acnes*, which may be located within the hair follicles and the pilosebaceous glands. This is the main bacterium responsible for causing surgical site infections, which is why we emphasize that the present work has identified new therapeutic options, where the mixture of extracts was able to promote reductions in the resistance structure of microorganisms (biofilm) with similar and even better results than the conventional antiseptic (CHX) in in vitro tests.

The antimicrobial and antibiofilm actions of the glycolic extracts of *R. officinalis *L., *P. paniculata*, and their combinations were tested in this study on planktonic and biofilm (monotypic and heterotypic) cultures of *S. aureus*, *S. epidermidis*, and *C. acnes*, where it was found that these extracts are effective. In addition, the same extracts were biocompatible with HaCaT without a statistically significant difference compared to the control group (DMEM + 10% FBS). Therefore, the null hypothesis of this study must be rejected.

To the best of our knowledge, this is the first study that evaluated the combined antimicrobial and antibiofilm actions of the glycolic extracts of *R. officinalis *L. and *P. paniculata* on *S. aureus* cultures, in which 100 mg/mL Mix B and Mix C resulted in a biofilm reduction of 82.68 and 80.77%, respectively. In the literature, Manilal et al. [19] evaluated the antimicrobial actions of the ethanolic, methanolic, and aceto-ethylic extracts of 100 mg/mL *R. officinalis* in planktonic and biofilm cultures of methicillin-resistant *S. aureus*, where the biofilm reduction varied from 71.4 to 85.7%. In the present study, the biofilm reduction of *S. aureus* cultures was of 78.56% with 100 mg/mL *R. officinalis* glycolic extract. In addition, the antimicrobial action of the glycolic extract of *R. officinalis* was tested on monomicrobial biofilms of *Candida albicans*, *Staphylococcus aureus*, *Enterococcus faecalis*, *Streptococcus mutans*, and *Pseudomonas aeruginosa* and on polymicrobial biofilms composed of *C. albicans* with each bacterium, where it was found that the extract at the concentration of 200 mg/mL was effective in reducing both monotypic and heterotypic biofilms [20].

In another study, the antimicrobial action of *R. officinalis* was tested on 29 aerobic and anaerobic bacteria and yeasts in the agar dilution test in which the extract was found to be effective on a variety of microorganisms, indicating its topic use for dermatologic disorders [21]. In this context, the essential oil of *R. officinalis* was effective on *C. acnes* by reducing the length, width, and height of the biofilm and altering the morphology of C. acnes, resulting in its death [22]. In the present study, *R. officinalis* when combined with *P. paniculata* in Mix B and Mix C was as effective as 0.12% CHX on *C. acnes*.

Moreover, the antimicrobial action of the aqueous extract of *R. officinalis* was effective over 34 aerobic and anaerobic bacteria, including *S. epidermidis*. In addition, the same study suggested the combination of *R. officinalis* aqueous extract with other herbal extracts like *Salvia rosmarinus*, *Salvia lavandulifolia*, and *Origan compactum* [23]. In the present study, the glycolic extract of *R. officinalis* at 100 mg/mL was the most effective group on *S. epidermidis*.

The glycolic extract of *P. paniculata* demonstrated antimicrobial action, promoting a great reduction in the heterotypic biofilm of *S. aureus*, *S. epidermidis*, and *C. acnes*, where the extract promoted a reduction of 91.78% of the biofilm structure that mimics the human skin microbiota. To the best of our knowledge, there are no similar studies in the literature to compare the results. However, Ramos et al. [16] demonstrated that the extract of *P. paniculata* promotes antimicrobial action against *Klebsiella pneumoniae* by reducing planktonic and biofilm cultures of the microorganism.

The application of the extracts on HaCaT resulted in percentages of viability greater than 50%. There was a statistically significant difference between the all the groups and the DMEM + 10% FBS control group (*p* < 0.05). In addition, the *R. officinalis* 100 mg/mL group was as biocompatible as the CHX group (*p* < 0.05). To the best of our knowledge, there are no studies that have evaluated the biocompatibility of *R. officinalis *L. and *P. paniculata* glycolic extracts on human keratinocytes; however, the biocompatibility of *R. officinalis *L. has been proven on a variety of human cells [15,24].

Finally, the isolated and combined extracts of *P. paniculata* and *R. officinalis* had antimicrobial effects on the planktonic and biofilm cultures of microorganisms from the skin microbiota, highlighting that the combinations of extracts promoted greater antimicrobial efficacy on monotypic and heterotypic biofilms, presenting similarly to, or even superiorly to, those obtained with CHX. It is also important to highlight that the three mixtures of extracts (Mix A, Mix B, and Mix C) promoted reductions of more than 90% on heterotypic biofilms of *S. aureus*, *S. epidermidis*, and *C. acnes.* This proves that it is a possible therapeutic option, exhibiting an antimicrobial effect on the human epithelial microbiota with low cytotoxic activity on human keratinocytes, thus being a new alternative to be explored in the asepsis of human skin.

## 4. Materials and Methods

### 4.1. Bacterial Strains and Plants Extracts

The reference strains of American Type Culture Collection of *Staphylococcus aureus* (ATCC 6538), *Staphylococcus epidermidis* (ATCC 12228), and *Cutibacterium acnes* (ATCC 6919) were used in microbiological analysis. A bacterial inoculum of each strain was prepared and adjusted by a spectrophotometer (Visible Spectrophotometer V-5000, Shanghai Metash Instruments Co., Ltd., Shanghai, China) in saline solution (NaCl 0.9%) (LGC Biotechnology^®^, Cotia, São Paulo, Brazil) at 1 × 10^6^ CFU/mL for MIC test and 1 × 10^8^ CFU/mL for biofilm test.

The extract of *Pfaffia paniculata* (Mapric Greentech company^®^, São Paulo, Brazil) was collected from the plant roots, and the extract of *Rosmarinus officinalis* (Mapric Greentech Company^®^, São Paulo, Brazil) was collected from the plant leaves. Both extracts were obtained commercially, diluted in propylene glycol in concentration of 20% (200 mg/mL).

### 4.2. Determination of Minimum Inhibitory and Minimum Bactericidal Concentration

Clinical and Laboratory Standards Institute (CLSI) protocol M7-A6 was followed to determine Minimum Inhibitory Concentration (MIC) and Minimum Bactericidal Concentration (MBC) using the microdilution broth method. The extracts *P. paniculata*; *R. officinalis*; 75% *P. paniculata* + 25% *R. officinalis* (Mix A); 50% *P. paniculata* + 50% *R. officinalis* (Mix B); and 25% *P. paniculata* + 75% *R. officinalis* (Mix C) were used in these analyses. In 96-well plates (TPP, Zollstrasse, Switzerland), 100 µL of Mueller Hinton Broth (Himedia^®^, Mumbai, India) was applied in each well, for a total of *n* = 10 for each group. Then, ten serial dilutions of 100 µL of each extract in the first well of the corresponding line of each plate were performed. The inoculum was prepared using microbial cultures with 24 h of incubation, using the following parameters: λ = 490 nm (*S. aureus* and *S. epidermidis*) with optical density (OD) = 0.374 ± 0.020 for both bacteria and λ = 600 nm and an optical density (OD) = 0.100 ± 0.020 for *C. acnes*. Then, 100 µL/well of each inoculum was added, respecting a different plate for each bacterial strain, for a total of 3 plates. All plates were incubated at 37 °C for 24 h (in anaerobic conditions for *C. acnes*). The MIC of each extract was determined considering the lowest concentration extract well that inhibited 50% of bacterial growth. For MBC determination, 10 µL of each well was inoculated in BHI agar (Himedia^®^, Mumbai, India) and incubated for 48 h at 37 °C (in anaerobic conditions for *C. acnes*). The lowest seeded concentration extract that did not show growth on solid medium was determined as the MBC.

In addition, the FIC was calculated using the formula FIC index = FIC_A_ + FIC_B_ = (MIC of extract A in combination/MIC of extract A alone) + (MIC of extract B in combination/MIC of extract B alone).

### 4.3. Monotypic and Cutaneous Model Biofilm

The bacterial inoculums were prepared again, following the same parameters described above, but using a concentration of 10^8^ CFU/mL, to form monotypic and heterotypic biofilms (cutaneous model biofilm). For monotypic biofilm, 100 µL of each respected bacterial inoculum and 100 µL of BHI broth (Himedia, Mumbai, India) were added to form biofilms in 3 days in 96-well plates (TPP, Zurich, Switzerland), using 8 wells from each line (*n* = 8) at 37 °C (in anaerobic conditions for *C. acnes*). For cutaneous biofilm model, 33.3 µL of each of the respective bacterial inoculum, totaling 100 µL of the three inoculums, and 100 µL of BHI broth (Himedia^®^, Mumbai, India) were added to form biofilms in 3 days in 96-well plates (TPP, Zollstrasse, Switzerland) using 8 wells from each line (*n* = 8) at 37 °C. The BHI broth (Himedia^®^, Mumbai, India) was changed every 24 h. Later, the biofilms were treated with two concentrations (50 and 100 mg/mL) of *R. officinalis*, *P. paniculata*, Mix A, Mix B, and Mix X for 24 h. In this analysis, BHI (Himedia^®^, Mumbai, India) and 0.12% CHX (Colgate-Palmolive^®^, New York, NY, USA) were used as control groups. Subsequently, the wells’ contents were discarded, and the biofilms were washed with sterile saline solution (LGC Biotechnology^®^, Cotia, São Paulo, Brazil) 3 times to remove dead cells.

### 4.4. Bacterial Viability by MTT Analysis

After the treatments, 100 µL of MTT solution 3-(4,5-Dimethyl-2-thiazolyl)-2,5-diphenyl-2H-tetrazolium bromide (CAS n°: 298-93-1, purity: 97.5%, Sigma-Aldrich^®^, St. Louis, MO, USA) was dispensed into each well of the plates, with subsequent incubation for 1 h at 37 °C (in anaerobic conditions for *C. acnes* monotypic and heterotypic biofilms). Then, MTT solution was discarded, and 100 µL of dimethyl sulfoxide (DMSO) (CAS n°: 67-68-5, purity: 99.9%, Sigma-Aldrich^®^, St. Louis, MO, USA) was added. The plates were incubated again for 10 min at 37 °C (in anaerobic conditions for *C. acnes* monotypic and heterotypic biofilms) and agitated by Shaker (Miulab^®^, Micro plate shaker MIX-1500, Hangzhou, China) for 10 min. Lastly, the plates were read at 570 nm on spectrophotometer (Lonza Biotek^®^, ELX808LBS, Winooski, VT, USA) to obtain the optical densities (OD) that were later converted into percentage of viability using the formula:

% Viability = (OD Treated Group × 100)/Mean OD Control Group)

### 4.5. Antimicrobial Analysis by Scanning Electron Microscopy

This analysis was carried out over heterotypic biofilms, which formed again as explained above, over polystyrene discs measuring 1 cm in radius and 0.5 cm in thickness deposited within 24 well plates (TPP, Zurich, Switzerland) (*n* = 2 for each experimental group). The treatments were applied for 24 h, and then the extracts were discarded and the biofilms were washed with sterile saline solution 0.9% (LGC Biotechnology^®^, Cotia, São Paulo, Brazil) with subsequent fixation with methanol (CAS n°: 67-56-1, purity: 99.8% Synth^®^, Diadema, Brazil) for 1 h. Then, the samples were gradually dehydrated with ethanol (CAS n°: 64-17-5, purity: 99.5%, Synth^®^, Diadema, Brazil) 10%, 25%, 50%, 75%, and 100% concentrations, and incubated at 37 °C for 24 h. The polystyrene discs were placed in aluminum stubs and covered with gold for 120 s at 40 mA (Quorum Technologies^®^, Emitech—SC7620, Kent, UK). Finally, the biofilms were analyzed by scanning electron microscope (FEI—Inspect S50, Hillsboro, OR, USA) in 4000×.

### 4.6. Human Keratinocytes Viability by MTT Analysis

The cells (HaCaT) were cultivated in Dulbecco’s Modified Eagle Medium (DMEM) (LGC Biotechnology, Cotia, São Paulo, Brazil) supplemented with 10% fetal bovine serum (FBS) (Invitrogen, New York, NY, USA), incubated at 37 °C, with atmospheric humidity, with 5% CO_2_ using cell culture flasks (TPP, Zurich, Switzerland). After reaching sub-confluence, which is characterized by 70% occupation of the cell culture flask, the cells were removed from the floor using trypsin (Sigma-Aldrich^®^, St. Louis, MO, USA) and sent for cell counting. The cytotoxicity was evaluated using a concentration of 4 × 10^4^ cell/well in 96-well plates (TPP, Zurich, Switzerland) incubated for 24 h for cellular adherence. The cells were treated with two concentrations (100 and 50 mg/mL) of *P. paniculata*, *R. officinalis*, Mix A, Mix B, and Mix C for 24 h. DMEM (LGC Biotechnology, Cotia, São Paulo, Brazil) + 10% FBS (Invitrogen, New York, USA) and 0.06% CHX (Colgate-Palmolive^®^, New York, NY, USA) were used as control groups. Then, 200 µL of MTT solution (CAS n°:298-93-1, purity: 97.5%, Sigma-Aldrich^®^, St. Louis, MO, USA) was added to each well and the plates were incubated for 1 h at 37 °C covered from light. Then, the solution was removed and 200 µL of DMSO (CAS n°: 67-68-5, purity: 99.9%, Sigma-Aldrich^®^, St. Louis, MO, USA) was added for 10 min at 37 °C and agitated by shaker (Miulab^®^, Micro plate shaker MIX-1500, Hangzhou, China) for 10 min. Lastly, the plates were read at 570 nm on spectrophotometer (Lonza Biotek^®^, ELX808LBS, Winooski, VT, USA) to obtain the optical densities that were later converted into percentage of viability using the formula:

% Viability = (OD Treated Group × 100)/Mean OD Control Group)

### 4.7. Statistical Analysis

Data were normally distributed by the D’Agostino, Shapiro-Wilk and Kolmogorov-Smirnov tests. They were statistically analyzed by the one-way ANOVA method complemented by the Tukey test, with a significance levels: *p* < 0.0332 (*), *p* < 0.0021 (**), *p* < 0.0002 (***), *p* < 0.0001 (****). Statistical analysis was carried out using GraphPad Prism 9.0 software.

## 5. Conclusions

The results of this study demonstrated that the combined *P. paniculata* and *R. officinalis* glycolic extracts have antimicrobial and antibiofilm effects on *S. aureus*, *S. epidermidis*, and *C. acnes*. In addition, these extracts are biocompatible against human keratinocytes. Therefore, these extracts represent an important and promising natural alternative in the fight against healthcare-associated infections.

## Figures and Tables

**Table 1:** MIC, MBC, and FIC values in mg/mL of *P. paniculata*, *R. officinalis*, and the combined extract on planktonic cultures of *S. aureus*, *S. epidermidis*, and *C. acnes*. (*P. paniculata* and *R. officinalis* 75% + 25% (Mix A), *P. paniculata* and *R. officinalis* 50% + 50% (Mix B), *P. paniculata* and *R. officinalis* 25% + 75% (Mix C), indifferent (IND), antagonistic (ANT), synergistic (SYN), not applicable (-)).

Extract	*S. aureus*			*S. epidermidis*			*C. acnes*		
	MIC	MBC	FIC	MIC	MBC	FIC	MIC	MBC	FIC
*P. paniculata*	25	25	-	25	Absent	-	6.25	50	-
Mix A	25	25	IND	50	Absent	ANT	3.12	25	SYN
Mix B	50	50	IND	50	Absent	ANT	3.12	25	SYN
Mix C	50	50	IND	50	Absent	ANT	6.25	6.25	IND
*R. officinalis*	50	Absent	-	25	Absent	-	0.195	6.25	-

**Table 2:** Evolution of *S. aureus* and *S. epidermidis* biofilm treated during 24 h by several mix of *P. paniculata* and *R. officinalis* by MTT analysis. (Brain Heart Infusion (BHI), *P. paniculata* and *R. officinalis* 75% + 25% (Mix A), *P. paniculata* and *R. officinalis* 50% + 50% (Mix B), *P. paniculata* and *R. officinalis* 25% + 75% (Mix C), chlorhexidine digluconate 0.12% (CHX 0.12%), (−) reduction on biofilm, (+) growing of biofilm, no significant (ns), *p* < 0.0332 (*), *p* < 0.0002 (***), *p* < 0.0001 (****). For statistical analyses all treatments were compared with BHI group).

Groups	*S. aureus*		*S. epidermidis*	
	% Evolution	*p* Value	% Evolution	*p* Value
Negative control (BHI)	+100		+100	
*P. paniculata* 50 mg/mL	−19.20	ns	−45.87	****
*P. paniculata* 100 mg/mL	−62.64	****	−76.64	****
*R. officinalis* 50 mg/mL	−46.63	***	−56.24	****
*R. officinalis* 100 mg/mL	−78.56	****	−80.11	****
Mix A 50 mg/mL	−18.46	ns	−79.13	****
Mix A 100 mg/mL	−79.10	****	−79.13	****
Mix B 50 mg/mL	−34.95	*	−78.38	****
Mix B 100 mg/mL	−82.68	****	−79.48	****
Mix C 50 mg/mL	−78.53	****	−77.65	****
Mix C 100 mg/mL	−80.77	****	−72.71	****
Positive control (CHX 0.12%)	−42.3	****	−74.9	****

**Table 3:** Evolution of *C. acnes* biofilm and heterotypic biofilms (in %) by MTT analysis after treatment by the groups for 24 h. (Brain Heart Infusion (BHI), *P. paniculata* and *R. officinalis* 75% + 25% (Mix A), *P. paniculata* and *R. officinalis* 50% + 50% (Mix B), *P. paniculata* and *R. officinalis* 25% + 75% (Mix C), chlorhexidine digluconate 0.12% (CHX 0.12%), (−) reduction on biofilm, (+) growing of biofilm, no significant (ns), *p* < 0.0332 (*), *p* < 0.0021 (**), *p* < 0.0002 (***), *p* < 0.0001 (****). For statistical analyses all treatments were compared with BHI group).

Groups	*C. acnes*		Heterotypic Biofilms	
	% Evolution	*p* Value	% Evolution	*p* Value
Negative control (BHI)	+100		+100	
*P. paniculata* 50 mg/mL	+260.60	****	−88.73	****
*P. paniculata* 100 mg/mL	+51.32	*	−91.78	****
*R. officinalis* 50 mg/mL	+17.97	ns	−90.90	****
*R. officinalis* 100 mg/mL	+48.32	ns	−92.82	****
Mix A 50 mg/mL	−46.33	ns	−93.46	****
Mix A 100 mg/mL	−56.36	*	−93.77	****
Mix B 50 mg/mL	+68.84	***	−92.77	****
Mix B 100 mg/mL	−36.26	ns	−93.50	****
Mix C 50 mg/mL	−36.26	ns	−92.78	****
Mix C 100 mg/mL	−47.31	ns	−91.78	****
Positive control (CHX 0.12%)	−50.5	**	−85.8	****

**Table 4:** Cellular viability of human keratinocytes (HaCaT) (in %) by MTT analysis after treatment by the groups for 24 h. (*P. paniculata* and *R. officinalis* 75% + 25% (Mix A), *P. paniculata* and *R. officinalis* 50% + 50% (Mix B), *P. paniculata* and *R. officinalis* 25% + 75% (Mix C), chlorhexidine digluconate 0.12% (CHX 0.12%), *p* < 0.0001 (****). For statistical analyses all treatments were compared with negative control group).

Groups	% Viability	*p* Value
Negative control (culture medium)	100	
*P. paniculata* 50 mg/mL	70.3	****
*P. paniculata* 100 mg/mL	60.1	****
*R. officinalis* 50 mg/mL	91.3	****
*R. officinalis* 100 mg/mL	60.1	****
Mix A 50 mg/mL	70.8	****
Mix A 100 mg/mL	59.7	****
Mix B 50 mg/mL	68.8	****
Mix B 100 mg/mL	59.7	****
Mix C 50 mg/mL	65.9	****
Mix C 100 mg/mL	59.1	****
Positive control (CHX 0.06%)	14.6	****

## Data Availability

The raw data supporting the conclusions of this article will be made available by the authors on request.

## References

[1] Rosenthal V.D., Yin R., Nercelles P., Rivera-Molina S.E., Jyoti S., Dongol R., Aguilar-De-Moros D., Tumu N., Alarcon-Rua J., Stagnaro J.P. (2024). International Nosocomial Infection Control Consortium (INICC) report of health care associated infections, data summary of 45 countries for 2015 to 2020, adult and pediatric units, device-associated module. Am. J. Infect. Control.

[2] Assis S.F., Vieira D.F.V.B., Sousa F.R.E.G., Pinheiro C.E.O., Prado P.R.D. (2022). Adverse events in critically ill patients: A cross-sectional study. Rev. Esc. Enferm. USP.

[3] Manoukian S., Stewart S., Dancer S., Graves N., Mason H., McFarland A., Robertson C., Reilly J. (2018). Estimating excess length of stay due to healthcare-associated infections: A systematic review and meta-analysis of statistical methodology. J. Hosp. Infect..

[4] Allegranzi B., Bagheri Nejad S., Combescure C., Graafmans W., Attar H., Donaldson L., Pittet D. (2011). Burden of endemic health-care-associated infection in developing countries: Systematic review and meta-analysis. Lancet.

[5] Li Pomi F., Papa V., Borgia F., Vaccaro M., Allegra A., Cicero N., Gangemi S. (2023). Rosmarinus officinalis and Skin: Antioxidant Activity and Possible Therapeutical Role in Cutaneous Diseases. Antioxidants.

[6] Elston M.J., Dupaix J.P., Opanova M.I., Atkinson R.E. (2019). *Cutibacterium acnes* (formerly *Proprionibacterium acnes*) and Shoulder Surgery. Hawai’i J. Health Soc. Welf..

[7] Anand S., Pogorelić Z., Singh A., Llorente Muñoz C.M., Krishnan N., Dhua A.K., Goel P., Bajpai M. (2022). Comparison of Unnoticed Glove Perforations during Minimally Invasive versus Open Surgeries: A Systematic Review and Meta-Analysis. Children.

[8] Amini Tapouk F., Nabizadeh R., Mirzaei N., Hosseini Jazani N., Yousefi M., Valizade Hasanloei M.A. (2020). Comparative efficacy of hospital disinfectants against nosocomial infection pathogens. Antimicrob. Resist. Infect. Control.

[9] Mehrad B., Clark N.M., Zhanel G.G., Lynch J.P. (2015). Antimicrobial resistance in hospital-acquired gram-negative bacterial infections. Chest.

[10] Chapman A.K., Aucott S.W., Milstone A.M. (2012). Safety of chlorhexidine gluconate used for skin antisepsis in the preterm infant. J. Perinatol..

[11] Roode G.J., Bütow K.W. (2018). A Descriptive Study of Chlorhexidine as a Disinfectant in Cleft Palate Surgery. Clin. Med. Res..

[12] Norman G., Christie J., Liu Z., Westby M.J., Jefferies J.M., Hudson T., Edwards J., Mohapatra D.P., Hassan I.A., Dumville J.C. (2017). Antiseptics for burns. Cochrane Database Syst. Rev..

[13] Rodenak-Kladniew B., Castro M.A., Gambaro R.C., Girotti J., Cisneros J.S., Viña S., Padula G., Crespo R., Castro G.R., Gehring S. (2023). Cytotoxic Screening and Enhanced Anticancer Activity of *Lippia alba* and *Clinopodium nepeta* Essential Oils-Loaded Biocompatible Lipid Nanoparticles against Lung and Colon Cancer Cells. Pharmaceutics.

[14] Atanasov A.G., Zotchev S.B., Dirsch V.M., Supuran C.T., International Natural Product Sciences Taskforce (2021). Natural products in drug discovery: Advances and opportunities. Nat. Rev. Drug Discov..

[15] de Oliveira J.R., Camargo S.E.A., de Oliveira L.D. (2019). *Rosmarinus officinalis* L. (rosemary) as therapeutic and prophylactic agent. J. Biomed. Sci..

[16] Paula-Ramos L., da Rocha Santos C.E., Camargo Reis Mello D., Nishiama Theodoro L., De Oliveira F.E., Back Brito G.N., Junqueira J.C., Jorge A.O.C., de Oliveira L.D. (2016). Klebsiella pneumoniae Planktonic and Biofilm Reduction by Different Plant Extracts: In Vitro Study. Sci. World J..

[17] Foster A.L., Cutbush K., Ezure Y., Schuetz M.A., Crawford R., Paterson D.L. (2021). *Cutibacterium acnes* in shoulder surgery: A scoping review of strategies for prevention, diagnosis, and treatment. J. Shoulder Elb. Surg..

[18] Dörfel D., Maiwald M., Daeschlein G., Müller G., Hudek R., Assadian O., Kampf G., Kohlmann T., Harnoss J.C., Kramer A. (2021). Comparison of the antimicrobial efficacy of povidone-iodine-alcohol versus chlorhexidine-alcohol for surgical skin preparation on the aerobic and anaerobic skin flora of the shoulder region. Antimicrob. Resist. Infect. Control.

[19] Manilal A., Sabu K.R., Shewangizaw M., Aklilu A., Seid M., Merdikios B., Tsegaye B. (2020). In vitro antibacterial activity of medicinal plants against biofilm-forming methicillin-resistant Staphylococcus aureus: Efficacy of *Moringa stenopetala* and *Rosmarinus officinalis* extracts. Heliyon.

[20] de Oliveira J.R., de Jesus D., Figueira L.W., de Oliveira F.E., Pacheco Soares C., Camargo S.E., Jorge A.O., de Oliveira L.D. (2017). Biological activities of *Rosmarinus officinalis* L. (rosemary) extract as analyzed in microorganisms and cells. Exp. Biol. Med..

[21] Weckesser S., Engel K., Simon-Haarhaus B., Wittmer A., Pelz K., Schempp C.M. (2007). Screening of plant extracts for antimicrobial activity against bacteria and yeasts with dermatological relevance. Phytomedicine.

[22] Fu Y., Zu Y., Chen L., Efferth T., Liang H., Liu Z., Liu W. (2007). Investigation of antibacterial activity of rosemary essential oil against *Propionibacterium acnes* with atomic force microscopy. Planta Medica.

[23] Boutahiri S., Eto B., Bouhrim M., Mechchate H., Saleh A., Al Kamaly O., Drioiche A., Remok F., Samaillie J., Neut C. (2022). *Lavandula pedunculata* (Mill.) Cav. Aqueous Extract Antibacterial Activity Improved by the Addition of *Salvia rosmarinus* Spenn., *Salvia lavandulifolia* Vahl and *Origanum compactum* Benth. Life.

[24] Manville R.W., Hogenkamp D., Abbott G.W. (2023). Ancient medicinal plant rosemary contains a highly efficacious and isoform-selective KCNQ potassium channel opener. Commun. Biol..

